# Therapeutic Potential of Human Amniotic Membrane-Derived Mesenchymal Stem Cell Conditioned Medium in Combating Oxidative Stress and Age-Related Female Infertility

**DOI:** 10.3390/cells14110801

**Published:** 2025-05-29

**Authors:** Kihae Ra

**Affiliations:** Department of Theriogenology and Biotechnology, College of Veterinary Medicine and Research Institute for Veterinary Science, Seoul National University, Seoul 08826, Republic of Korea; ragh1102@snu.ac.kr

**Keywords:** amniotic membrane-derived mesenchymal stem cell, conditioned medium, female reproductive system, oxidative stress, reproductive aging, RNA sequencing

## Abstract

Oxidative stress is a crucial factor accelerating the age-related functional deterioration of reproductive organs and fertility. Recently, human amniotic-membrane-derived mesenchymal stem cells (AMSCs) have emerged as a promising source with notable potential to reduce oxidative damage and support tissue regeneration. This study investigates the impact of the intravenous administration of human amniotic membrane-derived mesenchymal stem cell conditioned medium (AMSC-CM) on oxidative stress and reproductive competence in aged female mice. Antioxidative effects of human AMSC-CM were found in the reproductive organs of aged mice at both the RNA and protein levels. Human AMSC-CM positively regulated age-dependent changes in reproductive hormones, comparable to those observed in younger mice. RNA sequencing analysis revealed alterations in ovarian and uterine gene expression in aged mice showing that AMSC-CM treatment promotes the expression of genes essential for anti-aging, energy metabolism, and female reproductive processes. These findings highlight the potential of human AMSC-CM as a therapeutic strategy with anti-aging and antioxidative effects against age-related female infertility.

## 1. Introduction

Reproductive aging is assumed to be a major concern in terms of both the individual and socioeconomic aspects associated with infertility and adverse pregnancy-related complications. Age-related loss of ovarian and uterine function hampers the normal reproductive competence of aged females [1]. Female reproductive aging was once thought to be primarily caused by the deterioration of oocyte quality rather than uterine aging. However, increasing evidence indicates that both oocyte and uterine factors are involved in the decline in fertility in aged females [2]. Ovarian aging involves the gradual loss of ovarian follicles, a decrease in reproductive hormone production that is required for normal physiological functions, and the degradation of oocyte quality, all of which result in the decline in fertility [3]. The uterus, along with the ovaries, forms the essential reproductive organ where biological events for pregnancy occur, including embryonic development, elongation, implantation, placentation, and fetal development. The endometrial environment in the uterus determines the reproductive competence [4]. With aging, the number of implantation sites decrease during early pregnancy, and an abnormal uterine response disturbs the development of placenta decidualization, leading to a considerable reduction in fertility [5]. Age-dependent changes in the ovaries and uterus are known, together, to reduce the probability of a successful reproductive outcome [6]. Oxidative stress driven by excessive reactive oxygen species (ROS) increases with aging, bringing detrimental effects to essential ovarian and uterine functions necessary for fertility [7]. For instance, oxidative stress in the ovaries is linked to reproductive disorders, such as abnormal ovulation, polycystic ovary syndrome, and premature ovarian failure [8]. Oxidative damage disrupts progesterone production in the corpus luteum and its modulatory function in the endometrium, which are crucial for embryo development, implantation, and the maintenance of pregnancy [9]. Furthermore, an impaired immune system within the uterus caused by oxidative stress during gestation can potentially lead to early pregnancy loss [10]. Consequently, managing oxidative stress, particularly for aged females, is essential for maintaining successful reproductive outcomes.

Mesenchymal stem cell (MSCs) isolated from various source tissues have been widely used as a therapy to resolve age-associated reproductive disorders. Perinatal tissue including the placenta, amniotic membrane, and fluid, was considered to be unnecessary and was generally discarded post-partum; however, it is now acknowledged as a competent source of stem cells that have an intermediate stemness between pluripotency and multipotency [11,12,13]. The injection of human amniotic fluid-derived MSCs into the ovary has been shown to improve the function of aged ovaries, increase follicle numbers, and improve hormone levels in aged mice [14]. The intravenous injection (IV) of human umbilical cord-derived MSCs stabilizes the reproductive hormone levels and improves ovarian structure by upregulating genes involved in ovarian function in perimenopausal rats [15]. In mice showing natural ovarian aging, the injection of human amniotic membrane-derived MSCs (AMSC) into the ovary improves the number of ovarian follicles and the proliferation of ovarian granular cells, and inhibits apoptosis by increasing the secretion of growth factors [16].

The use of CM, the processed culture medium containing the secreted factors collected by culturing stem cells, offers significant advantages over the original cells in terms of safety and convenience [7]. A previous study demonstrated the anti-apoptotic and anti-oxidative effects of human ASC-CMs in the ovaries and uteruses of pregnant mice of advanced maternal age following IV administration under the optimal conditions [17]. However, studies were required to elucidate the undiscovered effects of other cell sources of human MSC-CM for advanced efficiency. As the findings of the previous study revealed the improved effects of AMSC-CM compared with that of ASC-CM on embryo development [18], this study further aimed to identify the physiological implications and potential mechanisms of AMSC-CM on the reproductive organs, with particular emphasis on its antioxidative and antiaging properties, by molecular-level analyses. Based on this, the present study aimed to investigate the changes in ovarian and uterine gene expression following the IV injection of human AMSC-CM using RNA sequencing analysis in association with its beneficial effects on oxidative stress and reproduction in aged female mice.

## 2. Materials and Methods

### 2.1. Ethics Approval

Human AMSCs were provided from the R Bio Stem Cell Research Center under GMP conditions. All of the cell donors provided informed consent for inclusion before participation in the study. The study was conducted in accordance with the Declaration of Helsinki, and the protocol was approved by the Ethics Committee of Biostar Stem Cell Technology (IRB NO. 2019-03).

### 2.2. Chemicals and Reagents

All chemicals and reagents were purchased from Sigma-Aldrich (St. Louis, MO, USA), unless stated otherwise.

### 2.3. Culture and Characterization of AMSCs

Isolation and establishment of AMSCs were performed as previously described [18]. Briefly, cryopreserved AMSCs (1 × 10^6^) from the amnion tissue of three donors were thawed and seeded in T-175 flasks (175 cm^2^) containing RPME-P (R BIO, Seoul, Republic of Korea) supplemented with an antibiotic–antimycotic solution (1%) in a humidified incubator at 37 °C and 5% CO_2_. AMSCs were cultured in AMSC medium (R BIO) until the cells were confluent. The immunophenotypic characterization of cultured AMSCs was performed using flow cytometry. The method and result of characterization were previously stated [18].

### 2.4. Preparation of AMSC-CM

AMSCs from a donor were cultured in RPME-P until 90% confluency, and then the medium was changed to Dulbecco’s modified Eagle’s medium (DMEM) after washing twice with PBS. The culture medium was collected every 24 h, and fresh DMEM was added to the original flask. Supernatants were collected for 5 d and then pooled. The pooled medium was centrifuged at 1700 rpm for 5 min and filtered using a 0.22 µm filter to be prepared as CM. CM from three donors was equally mixed in a total volume of 20 mL and concentrated to a final volume of 2 mL by centrifugation at 3000× *g* for 90 min at 4 °C using a 3 kDa cut-off filter tube (Vivaspin 20; GE healthcare, Chicago, IL, USA).

### 2.5. Experimental Animals

The 7- and 28-week-old female C57BL/6J mice (Central Lab Animal Inc., Seoul, Republic of Korea) were housed under conventional conditions in an animal facility at the Seoul National University. Experimental animals were kept in a room under controlled temperature (23 °C) and humidity conditions (60%) with a 12 h light/dark cycle. Mice were humanely sacrificed by cervical dislocation as required. All experimental animal procedures were approved by the Institutional Animal Care and Use Committee of Seoul National University (SNU-200928-2-2).

### 2.6. Intravenous Administration of AMSC-CM

The 7-week-old mice were categorized into the ‘young control’ group (YC) and 28-week-old mice were randomly divided into the ‘old control’ (OC) and ‘old treatment’ (OT) groups. The OT group was intravenously injected with human AMSC-CM via the tail vein six times with 4 d intervals, which was previously confirmed as the optimal condition for IV CM treatments [17]. The YC and OC groups were intravenously injected with normal saline (0.9%) (JW Pharmaceutical, Seoul, Republic of Korea). The single dose amount was determined based on the weight of each mouse (1 μL/g). Prior to the treatment, pregnant mare serum gonadotropin (10 IU), followed by human chorionic gonadotropin (10 IU) after 48 h, were intraperitoneally injected into every group for estrous cycle synchronization.

### 2.7. Oxidative Stress and Antioxidant Marker Assays

The ovaries and uterine tissue lysates from the YC, OC, and OT groups were used to measure total antioxidant capacity (TAC), hydrogen peroxide (H_2_O_2_), and catalase (CAT) activities using OxiSelect™ assay kits (Cell Biolabs Inc., San Diego, CA, USA) according to the manufacturer’s instructions. The collected ovaries and uterine tissue samples were homogenized and centrifuged, and the supernatant was collected according to the manufacturer’s protocol, as per the recommendations in the respective assay kit. The results of each colorimetric assay were calculated using absorbance values measured at 490 nm for TAC activity, 520 nm for CAT activity, and 570 nm for H_2_O_2_ activity.

### 2.8. Serum Hormone Measurement

Serum was collected from mice in the YC, OC, and OT groups, and the levels of follicle-stimulating hormone (FSH) and anti-mullerian hormone (AMH) were measured using an enzyme immunoassay kits (Mybiosource, San Diego, CA, USA) according to the manufacturer’s instructions. The optical density of the samples was measured using a spectrophotometer at 450 nm.

### 2.9. RNA Sequencing Analysis

RNA sequencing was performed on the ovaries and uterine tissues collected from the mice in the OC (*n* = 3) and OT (*n* = 4) groups. The RNA library was prepared using an Illumina TruSeq Stranded Total RNA Sample Prep Kit (Illumina, San Diego, CA, USA) according to the manufacturer’s instructions. Briefly, after removing rRNA from the total RNA (700 ng) using an rRNA removal kit, RNA was purified using beads. After rRNA depletion, a fragment mix (EPH) was used for the remaining RNA at 94 °C for 6 min. Fragmented RNA primed with random hexamers was transcribed to first-strand cDNA using reverse transcriptase and random primers at 25 °C for 10 min, 42 °C for 15 min, and 70 °C for 15 min. Subsequently, to produce double-stranded cDNA, the replacement strand was synthesized by incorporating dUTP in place of dTTP using polymerase at 16 °C for 1 h. After cDNA was cleaned using sample purification beads, a single “A” nucleotide was attached to the 3′ ends of the blunt fragments using the A-Tailing mix reagent by incubation at 37 °C for 30 min, and then at 70 °C for 5 min. Indexing adapters were ligated to the ends of the DNA fragments using ligation mix 2 reagents at 30 °C for 10 min. PCR was conducted to enrich the DNA fragments with adapter molecules on both ends after washing twice with sample purification beads under the thermocycler conditions, as follows: 95 °C for 3 min, followed by eight cycles of 98 °C for 20 s, 60 °C for 15 s, and 72 °C for 30 min, with a final extension at 72 °C for 5 min. The quality and band size of the libraries were evaluated using an Agilent 2100 Bioanalyzer (Agilent, Santa Clara, CA, USA). Libraries were quantified by qPCR using the CFX96 Real-Time System (Bio-Rad, Hercules, CA, USA) and normalized. Sequencing of the prepared library was conducted using the NextSeq 500 system (Illumina). Potential sequencing adapters in the raw reads were trimmed using Skewer (version 0.2.2). The trimmed reads were mapped to the reference genome using STAR (version 2.5) software [19]. Since the sequencing libraries were strand specific, prepared using Illumina’s strand-specific library preparation kit, the mapping process was performed using the strand-specific library option (--library-type = fr-firststrand). Cufflinks software (version 2.2.1) [20] was used with the strand-specific library option (--library-type = fr-firststrand) and other default options for the quantification of the mapped reads on the reference genome to the gene expression values. The gene annotation of the reference genome (mm10) from UCSC Genome Browser (https://genome.ucsc.edu) in the GTF format was used as a gene model, and the expression values were calculated in the fragments per kilobase of transcript per million fragments mapped (FPKM) unit. Differentially expressed genes (DEGs) between the OC and OT groups were analyzed using Cuffdiff software in the Cufflinks package [21] using the strand-specific library option (--library-type = fr-firststrand) and other default options. The normalized expression values of the selected DEGs were clustered unsupervised and scatter plots for the gene expression values between the two groups were drawn using in-house R scripts to compare the expression profiles.

### 2.10. Functional Category Analysis

To investigate the physiological function of the DEGs in the ovarian and uterine tissues between the OC and OT groups, the gene set overlapping test was conducted between the analyzed DEGs and functional categorized genes regarding biological processes of Gene Ontology by g: Profiler version 0.6.7 [22]. Gene set enrichment analysis was performed using the GSEA software v4.1.0 (https://www.gsea-msigdb.org/gsea/index.jsp, accessed on 11 August 2020).

### 2.11. Statistical Analyses

Statistical analyses were performed using GraphPad Prism version 5 (GraphPad; San Diego, CA, USA) using a one-way ANOVA followed by Tukey’s post-hoc test. Data are presented as the mean ± standard error of the mean. A *p*-value of less than 0.05 was considered statistically significant among the groups. All experiments were conducted in at least three replicates.

## 3. Results

### 3.1. AMSC-CM Treatment Restrains Oxidative Stress and Improves Antioxidant Levels

To evaluate the oxidative effect of AMSC-CM administration in the reproductive organs of aged female mice, oxidative stress and antioxidant biomarker levels were compared in the ovarian and uterine lysates from the YC, OC, and OT groups.

**Figure 1:**
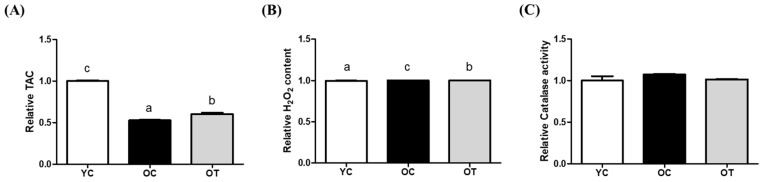
Comparison of oxidative stress and antioxidant biomarker levels in ovaries of young control (YC), old control (OC), and old treatment groups injected with AMSC-CM. The relative total antioxidant capacity (TAC) (**A**), hydrogen peroxide (H_2_O_2_) content (**B**), and catalase activity (**C**) were expressed. Data are normalized to average value of ASC-CM and presented as the mean ± SEM. Different superscript letters in each column indicate significant difference (*p* < 0.05).

In the ovarian lysates, the TAC of the OT group was significantly higher than that of the OC group (*p* < 0.01), but was lower than that of the YC group (*p* < 0.001, Figure 1A). The H_2_O_2_ content of the OT group was significantly decreased compared to OC group, but not as compared to the YC group (*p* < 0.001, Figure 1B). CAT activity showed no differences among all of the groups (Figure 1C).

**Figure 2:**
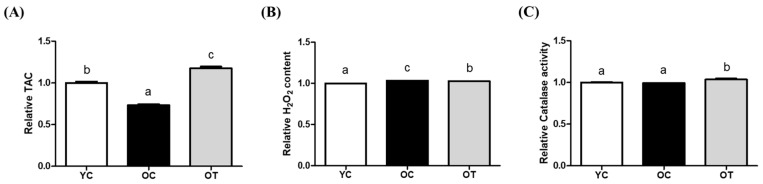
Comparison of oxidative stress and antioxidant biomarkers level in uterus of young control (YC), old control (OC), and old treatment groups injected with AMSC-CM. The relative total antioxidant capacity (TAC) (**A**), hydrogen peroxide (H_2_O_2_) content (**B**), and catalase activity (**C**) were expressed. Data are normalized to average value of ASC-CM and presented as the mean ± SEM. Different superscript letters in each column indicate significant difference (*p* < 0.05).

In the uterine lysate, the TAC of the OC group was the lowest (*p* < 0.001), and that of the OT group was significantly improved compared to the YC (*p* < 0.001) and the OC groups (*p* < 0.001, Figure 2A). The H_2_O_2_ content of the OT group was significantly lower than that that of the OC group, but not higher than that of the YC group (*p* < 0.0001, Figure 2B). However, in contrast to the results with the ovarian lysate, the CAT activity was significantly increased in the OT group compared with both the YC (*p* < 0.05) and OC groups (*p* < 0.01, Figure 2C).

### 3.2. AMSC-CM Treatment Restores Serum Reproductive Hormone Levels

**Figure 3:**
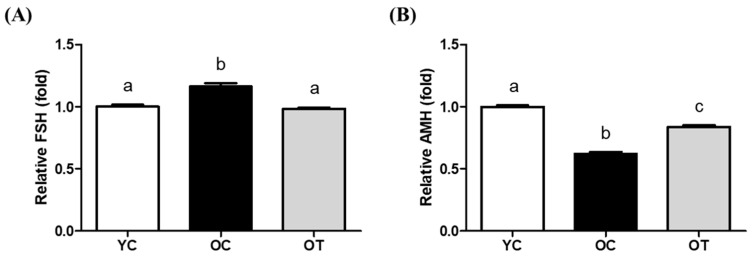
Relative serum hormone levels in young control (YC), old control (OC), and old treatment (OT) groups injected with AMSC-CM. (**A**) Follicle stimulating hormone (FSH); (**B**) anti-mullerian hormone (AMH). Data are presented as the mean ± SEM. Different superscript letters in each column indicate significant difference (*p* < 0.05).

In addition to antioxidation, the physiological effects of AMSC-CM administration in aged female mice reproduction were validated by the serum hormone levels of FSH and AMH. The serum levels of FSH were significantly higher in the OC group than in the YC and OT groups (*p* < 0.01). FSH levels in the YC and OT groups were similar (Figure 3A). AMH levels in the OC and OT groups were lower than in the YC group but significantly increased in the OT group compared to the OC group (*p* < 0.001, Figure 3B).

### 3.3. AMSC-CM Treatment Alters the Ovary and Uterus Transcriptomes

**Figure 4:**
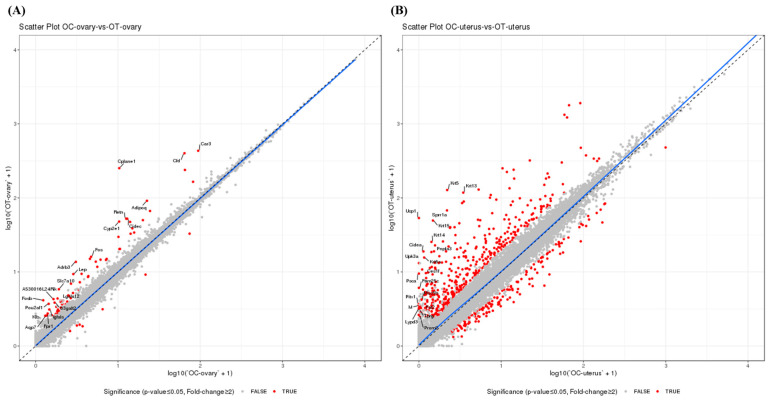
Scatter plot based on gene expression profiles to show the similarities among samples. Differentially expressed genes between groups were identified using a fold-change cutoff of 2 and a *p*-value cutoff of 0.05. (**A**) OC-group ovaries vs. OT-group ovaries; (**B**) OC-group uteruses vs. OT-group uteruses.

For molecular understanding of the antioxidative and physiological effects above, transcriptome analysis was conducted in the ovaries and uterus of aged female mice according to AMSC-CM administration. The quality of reads from each sample was assessed by the rate of reads whose sequencing quality was over 30 in the total number of reads. The percentage of samples showing over Q30 bases was higher than 80%, thus validating the read quality. The normalized distribution of transcript expression in each sample of the OC and OT groups was identical. DEGs between the OC and OT groups were identified using a fold-change cutoff of 2 and a *p*-value cutoff of 0.05. Among a total of 75 DEGs, the unsupervised hierarchical clustering analysis found that 67 genes were upregulated and eight genes were downregulated in the OT-group ovaries compared to that in the OC-group ovaries (Figure 4A). As compared to the OC-group uteruses, 431 genes were upregulated and 163 genes were downregulated among a total of 594 DEGs (Figure 4B) in the OT-group uteruses (Figure 4B). Additionally, 54 of the 75 ovarian DEGs overlapped with the uterine DEGs between the OC and OT groups. Except for three genes, all genes were upregulated in both the ovary and uterus (Table 1).

### 3.4. Functional Analysis of the Ovaries in Singular Enrichment Analysis Terms

**Figure 5:**
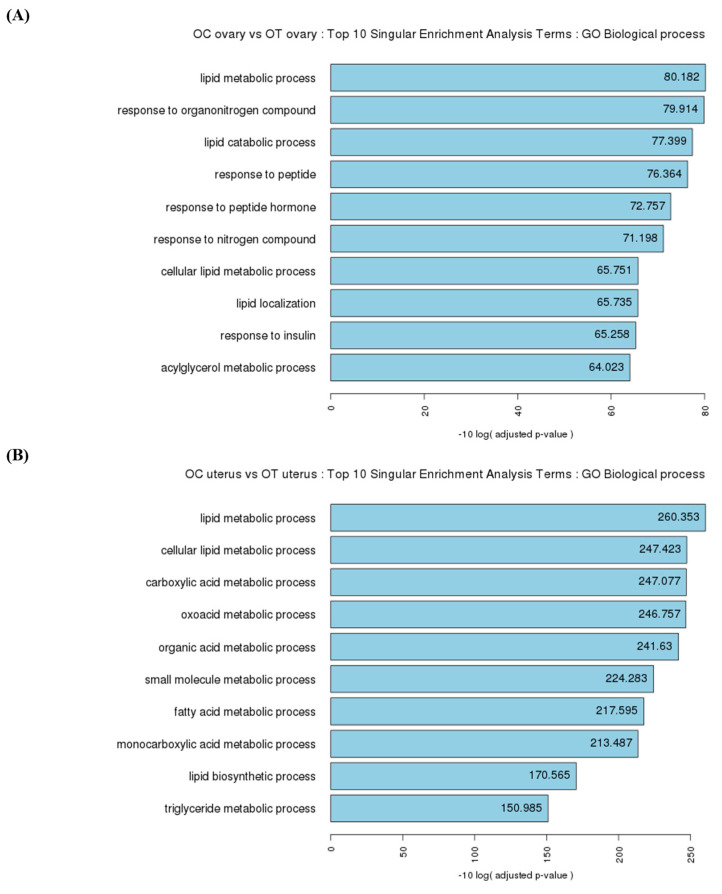
Top 10 singular enrichment analysis terms in GO biological process. (**A**) OC-group ovaries vs. OT-group ovaries, the enriched biological terms from the 75 DEGs selected by a fold-change cutoff of 2 and a *p*-value cutoff of 0.05. (**B**) OC-group uteruses vs. OT-group uteruses, the enriched biological terms from the 594 DEGs selected by a fold-change cutoff of 2 and a *p*-value cutoff of 0.05.

Enriched Gene Ontology (GO) terms in the biological process category were analyzed using the g:Profiler (version 0.6.7) R package. In the biological process, 114 terms were significantly enriched in the OT-group ovaries compared to those in the OC group, including response to stimulus, immune system process, metabolic process, homeostatic process, and female pregnancy. As is shown in Figure 5A, the top ten enriched terms were lipid metabolic process, response to organonitrogen compound, lipid catabolic process, response to peptide, response to peptide hormone, response to nitrogen compound, cellular lipid metabolic process, lipid localization, response to insulin, and acylglycerol metabolic process.

### 3.5. Functional Analysis of the Uterus in Singular Enrichment Analysis Terms

Among the enriched GO terms in biological processes, 348 terms were significantly enriched in the OT-group uteruses compared to those in the OC group, including cell differentiation, structure development, regulation of peptide secretion, response to stimulus, metabolic process, and homeostatic process. As is shown in Figure 5B, the top ten enriched terms were lipid metabolic process, cellular lipid metabolic process, carboxylic acid metabolic process, oxoacid metabolic process, organic acid metabolic process, small molecule metabolic process, fatty acid metabolic process, monocarboxylic acid metabolic process, lipid biosynthetic process, and triglyceride metabolic process.

### 3.6. Gene Set Enrichment Analysis of the Ovaries

**Figure 6:**
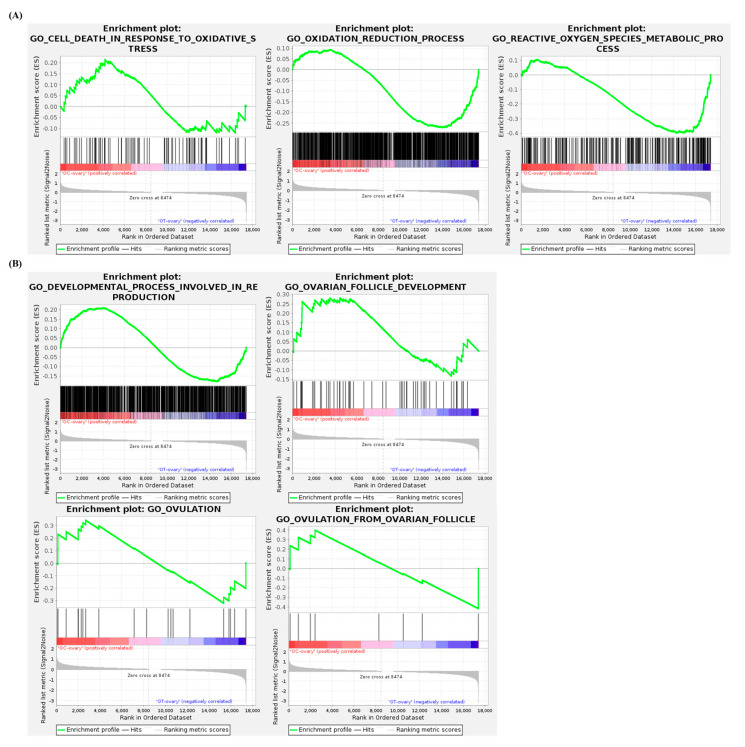
Gene set enrichment analysis between OC-group ovaries and OT-group ovaries. GO terms related to oxidative stress (**A**) and reproductive processes (**B**) in ovaries were presented.

GSEA was performed to compare the gene enrichment of selected terms in the ovaries of the OC and OT groups. The list of terms analyzed (Figure 6A,B) include the following: cell death in response to oxidative stress (normalized enrichment score: 0.814224, nominal *p*-value: 0.912), oxidation reduction process (−1.1907881, 0.026286965), reactive oxygen species metabolic process (−1.6204555, 0), developmental process involved in reproduction (1.0326562, 0.29069766), ovarian follicle development (0.99881315, 0.4673913), ovulation (0.96755165, 0.5), and ovulation from ovarian follicles (−0.8856735, 0.6181172).

### 3.7. Gene Set Enrichment Analysis of the Uterus

**Figure 7:**
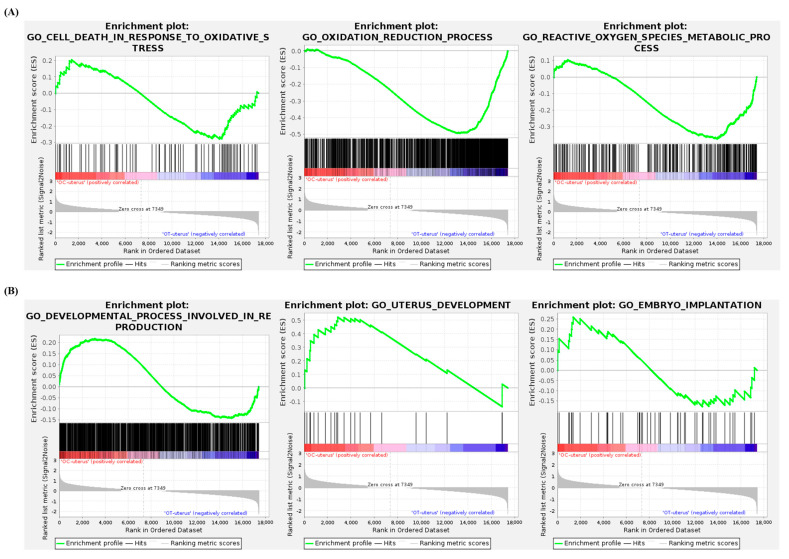
Gene set enrichment analysis between OC-group uteruses and OT-group uteruses. GO terms related to oxidative stress (**A**) and reproductive process (**B**) in uteruses were presented.

As is shown in Figure 7A,B, the list of terms analyzed by GSEA between the uteruses of the OC and OT groups include the following: cell death in response to oxidative stress (normalized enrichment score: −1.0534321, nominal *p*-value: 0.35905045), oxidation reduction process (−2.3681388, 0), reactive oxygen species metabolic process (−1.6318362, 0.0012919897), developmental process involved in reproduction (1.176085, 0), uterine development (1.5872613, 0.017721519), and embryo implantation (0.9732308, 0.4863222).

## 4. Discussion

In this study, the ovarian and uterine gene expression changes in aged mice were identified following the IV administration of AMSC-CM, which could demonstrate antioxidative and physiological effects in reproduction. These findings build on the previously reported antioxidant effect of AMSC-CM [18], thereby confirming the beneficial effects of AMSC-CM on female reproduction. First of all, oxidative stress and antioxidant biomarkers in the ovarian and uterine lysates were measured to confirm the antioxidative effect of AMSC-CM at the protein level. As there is a cumulative ability of antioxidants to counteract oxidative stress, TAC provides an overall measure of the antioxidant defense system maintaining cellular functions and preventing damage caused by ROS in various physiological and pathological conditions [23]. The TAC of reproductive organs in aged mice was lower than that in the young mice, as predicted; but, remarkably, AMSC-CM treatment improved the TAC in both the ovary and uterus (Figure 1A and Figure 2A). In particular, the TAC of old mice uteruses treated with AMSC-CM was observed to be higher than that of YC mice (Figure 2A). The contents of H₂O₂ in both the ovary and uterus were reduced after the IV administration of AMSC-CM to aged mice (Figure 1B and Figure 2B), showing its antioxidative effects on a key ROS that contributes to oxidative stress by causing damage to cellular components, including DNA, proteins, and lipids, associated with pathological conditions such as inflammation and aging [24]. Of note, the activity of CAT, which was one of the DEGs upregulated specifically in the OT group (in the uterus), was enhanced after AMSC-CM treatment in aged mice (Figure 2C). Moreover, this is consistent with the previous finding that adipose-derived MSC (ASC)-CM treatment as conventional antioxidant interventions in aged female mice increased CAT expression in the uterus by 7-fold in 4-month-old mice and 1.5-fold in 6-month-old mice [25].

Next, the reproductive hormones, namely FSH and AMH levels, were evaluated in the serum of the YC, OC, and OT groups. FSH and AMH are markers of aging as an increase in the FSH level and a decrease in the AMH level indicate ovarian aging [26], depletion of the follicular reserve, and uterine atrophy [27]. While aged mice showed increased FSH levels (Figure 3A), AMSC-CM treatment reduced FSH levels in aged mice, similar to the levels in the YC mice. Furthermore, as a biomarker to assess reproductive age [28], the AMH level was upregulated in aged mice by AMSC-CM treatment (Figure 3B). As the YC, OC, and OT groups were equally injected with superovulation-induction hormones for estrous cycle synchronization, the possibility that the difference in the hormone levels among groups is due to individual estrous cycles can be excluded. Thus, the results demonstrate the anti-aging effect of AMSC-CM on age-dependent changes in serum hormone levels.

In the transcriptome analysis, among the 54 genes differentially expressed in common in the AMSC-CM-treated ovaries and uteruses of aged mice compared to the control group (Table 1), the most remarkable genes were klotho beta (*klb*) and *adipoq*. Klotho has been identified as an aging-suppressor gene, and the its overexpression in mice leads to an extension of lifespan by 20–30% through regulation of the aging process [29]. Klotho helps to preserve cellular health during aging by supporting redox balance and counteracting oxidative stress, thereby contributing to mitochondrial function and overall metabolic stability [30,31,32]. *klb*, as a crucial element of endocrine fibroblast growth factor (FGF) receptor complexes, controls multiple metabolic processes including energy metabolic processes, glucose uptake, and fatty acid metabolism [29,33]. Interacting with FGF signaling, recent studies have demonstrated that *klb* functions as a critical molecular integrator of metabolism and reproductive aging. Its expression is associated with both hypothalamic gonadotropin-releasing hormone signaling and peripheral redox balance, linking systemic metabolic cues to reproductive axis regulation and ovarian homeostasis [34,35]. In the present study, treatment with AMSC-CM led to a marked upregulation of *klb* expression in the aged ovary and uterus, suggesting AMSC-CM may regulate the metabolic–reproductive crosstalk that is disrupted by aging, and its enhancement of *klb*-associated signaling could be a potential strategy for restoring age-related reproductive decline through both metabolic and redox-mediated mechanisms.

*Adipoq* encodes the protein adiponectin, one of the adipokine hormones that act as critical regulators in female reproductive organs regarding ovarian steroidogenesis, oocyte maturation, development, and the embryo implantation environment [36]. The effect of adiponectin on the ovary includes the activation of ovarian follicle remodeling genes, oocyte maturation, corpus luteum development, and the secretion of ovarian hormones [37]. Furthermore, adiponectin affects the uterine expression of genes involved in uterine tissue development, proliferation, apoptosis, and the regulation of implantation [37]. The interaction of *adipoq* and its receptors is associated with preimplantation embryo development and uterine receptivity through autocrine and paracrine signaling [38]. Mechanistically, *adipoq* plays a critical role in oxidative regulation, a key contributor to reproductive aging, by activating the AMP-activated protein kinase (*AMPK*) signaling pathway [36,37,38]. *AMPK* serves as an upstream modulator of the Forkhead box O (*FoxO*) transcription factors and their downstream genes involved in oxidative stress resistance, ultimately modulating cellular antioxidant defense [39,40,41]. Interestingly, in the previous study, *FoxO*-mediated expression of antioxidant genes were increased in blastocysts that developed in vitro in AMSC-CM–supplemented culture medium [18]. While the previous study focused on preimplantation embryos and the present study investigates ovarian and uterine tissues in the context of reproductive aging, the shared upregulation of genes involved in *AMPK* pathway indicates a broad anti-oxidative effect of AMSC-CM on reproductive competence. Taken together, these findings suggest that AMSC-CM may modulate oxidative stress not only in somatic reproductive tissues but also throughout the oocyte-to-blastocyst developmental trajectory via the *Adipoq*–*AMPK*–*FoxO* axis.

In both ovarian and uterine genes, the top ten singular enrichment analysis terms in biological processes, between the AMSC-CM-untreated and -treated groups, were primarily categorized under metabolic processes (Figure 5A,B). Among the various metabolic processes, energy metabolism, a common function of *klb* and *adipoq*, declines progressively with age [42]. The resulting metabolic problems may induce reproductive disorders, including ovarian disease or infertility [43]. The lipid-metabolism-related terms were predominant in the top ten singular enrichment analysis terms (Figure 2B). Lipid metabolism is highly involved in the regulation of aging, as demonstrated by studies showing that interventions modulating lipid metabolism increase life expectancy [44]. In the aspect of reproduction, abnormal lipid metabolism is a leading risk factor for infertility in aged women [45]. In the perimenopausal state, the administration of antioxidants can be applied to modulate disrupted lipid metabolism and improve ovarian function [46]. Furthermore, a disorder of lipid metabolism is considered to induce an adverse pregnancy outcome, considering the essential roles of lipids in various cellular processes, including cell apoptosis, differentiation, and growth [47]. In particular, uterine lipid metabolism is critical for uterine receptivity [48]. It was hypothesized that the IV administration of AMSC-CM in aged mice would alleviate aging-induced damage to reproduction and oxidative stress. Indeed, this study found that the genes related to female pregnancy were differentially expressed in the OT-group ovaries compared to those in the OC group. Moreover, based on the analysis of the GO biological process terms, the genes related to detoxification, the defense response, the oxidation–reduction process, and the reactive oxygen species metabolic process were differentially expressed in the OT-group uteruses compared to those in the OC group. Further, GSEA for specific terms was performed to evaluate the effect of AMSC-CM treatment on the ovaries and uteruses of aged mice, focusing on reproduction and oxidative stress. GSEA suggested that genes involved in the regulation of oxidation were enriched in the uterus following AMSC-CM treatment compared to those in the control group (Figure 7). In addition to oxidation reduction and ROS metabolism, genes engaged in ovulation were also enriched in the ovaries of the treatment group as compared to those of the control group (Figure 6).

These findings bridge the molecular effects of AMSC-CM with functional improvements in antioxidant modulation and endocrine regulation, providing a robust foundation for future applications to age-related infertility. Although the present study remains at the preclinical stage, existing findings suggest that AMSC-CM could be a promising therapeutic agent and an effective solution for female reproductive aging, advancing from bench to bedside [7,18]. As recent studies have pointed out, AMSC-CM has emerged as a potential candidate with advantages over cell-based approaches in terms of safety and practical aspects, such as being free from immunogenicity risks, having a standardized production protocol, and offering storage stability and manageability. From a legal standpoint, regulatory guidelines for manufacturing and clinical trial standards will be required to establish AMSC-CM as a translational medicine after thorough preclinical research investigating its pragmatic applications. In this regard, expanding on the outcomes of the present study, further research could evaluate additional metrics of reproductive performance, such as implantation rates and live birth outcomes, to provide a more comprehensive understanding of functional improvements. Additionally, identifying specific factors within AMSC-CM that contribute to these antioxidative and anti-aging effects would clarify the underlying mechanisms and support its therapeutic applications.

## 5. Conclusions

The IV of human AMSC-CM resulted in upregulated genes with their main functions being modulating aging and endocrine effects in the female reproductive system and biological metabolic processes in the ovaries and uteruses of aged female mice. Moreover, the antioxidative effect of human AMSC-CM was confirmed at both the RNA and protein levels in the ovary and uterus as the level of indicative markers of antioxidant capacity was recovered and age-dependent alteration of reproductive hormones was restored, all of which resulted in the improvement of reproductive competence in aged mice.

## Figures and Tables

**Table 1:** Differentially expressed genes that are commonly upregulated in the ovary and uterus between the OC and OT groups.

OC vs. OT					
Official Gene Symbol	Log_2_ (Fold Change)		Official Gene Symbol	Log_2_ (Fold Change)	
	Ovary	Uterus		Ovary	Uterus
*Slc7a10*	2.406	5.256	*Ffar2*	1.260	3.868
*A530016L24Rik*	2.35	5.092	*Lpl*	1.362	3.758
*Cfd*	2.66	4.759	*Rbp4*	1.689	3.423
*Adrb3*	2.61	4.793	*Fosb*	3.741	1.284
*Klb*	2.35	5.009	*Retnla*	1.219	3.601
*Pnpla3*	1.819	5.391	*Abcd2*	1.184	3.334
*Pou2af1*	2.623	4.517	*Trarg1*	1.421	3.089
*Aqp7*	2.163	4.924	*Cdo1*	1.829	2.349
*Cyp2e1*	2.327	4.736	*Cd36*	1.48	2.575
*Car3*	2.211	4.396	*Irs3*	1.481	2.518
*Cidec*	2.102	4.377	*Jchain*	1.653	2.341
*Plin1*	1.438	4.895	*Fcor*	1.21	2.779
*Retn*	2.147	4.182	*Ces1d*	1.022	2.706
*Fabp4*	1.889	4.293	*Mzb1*	1.359	2.265
*B3galt2*	2.034	4.098	*Cd79b*	1.544	2.017
*Lgals12*	2.000	4.037	*Acp5*	1.14	2.398
*Adipoq*	2.071	3.909	*Ifi27l2a*	1.022	2.368
*Adig*	1.407	4.516	*Hcar1*	1.023	2.289
*Lep*	2.163	3.73	*Fos*	2.016	1.125
*Acvr1c*	1.483	4.181	*Cd209g*	1.029	1.871
*Pck1*	1.545	4.068	*Pxmp2*	1.279	1.493
*Apoc1*	1.154	4.404	*Tmem45b*	1.023	1.676
*Igfals*	2.183	3.335	*Ccl2*	1.284	1.384
*Orm1*	1.36	4.096	*Dgat2*	1.166	1.411
*Serpina3c*	1.284	4.144	*Thrsp*	1.265	1.218
*Myl1*	1.039	4.181	*Pla2g2d*	1.066	1.218
*Sncg*	1.979	3.201			

## Data Availability

The RNA sequencing data presented in this study are available in the Gene Ex-pression Omnibus (GEO) under the accession number GSE297514.

## Abbreviations

The following abbreviations are used in this manuscript:

MSCs	Mesenchymal stem cells
AMSCs	Amniotic membrane-derived mesenchymal stem cells
CM	Conditioned medium
ROS	Reactive oxygen species
IV	Intravenous injection
EGs	Differentially expressed genes
FSH	Follicle-stimulating hormone
AMH	Anti-mullerian hormone
TAC	Total antioxidant capacity
SOD	Superoxide dismutase
CAT	Catalase
GSEA	Gene set enrichment analysis
